# How to do it: the difficult thyroid

**DOI:** 10.1186/1758-3284-3-54

**Published:** 2011-12-23

**Authors:** Tahwinder Upile, Waseem Jerjes, Jaspal Mahil, Hitesh Tailor, Ramkishan Balakumar, Anuja Rao, Yassar Qureshi, Iain Bowman, Suchana Mukhopadhyay

**Affiliations:** 1 Department of ENT/Head and Neck Surgery, Barnet and Chase Farm Hospitals NHS Trust, Enfield, UK; 2 Head and Neck Unit, University College London Hospitals, London, UK; 3 Dept. of Surgery, University College London Medical School, London, UK; 4 Leeds Institute of Molecular Medicine, School of Medicine, University of Leeds, Leeds, UK; 5 Oral and Maxillofacial Surgery Unit, AL-Mustansirya University, Baghdad, Iraq

## Abstract

There is a paucity of publications detailing how to deal with the difficult thyroid cancer. When compared to other cancers, it is relatively rare with several histopathological subtypes which run differing clinical courses and respond to different therapies. It is a condition predominately treated by specifically trained General and now ENT surgeons who already have a thorough knowledge of vocal fold assessment and rehabilitation as well as emergency airways management both to avoid and treat common complications should they occur.

Good surgery involves a team effort to produce good results consistently. All members of the team are essential to quality service delivery. Communication with the team and the patient is paramount. We describe our approach to the difficult thyroid.

## Introduction

### Thyroid disease

Most thyroid nodules are benign and more often occur in females. Thyroid cancer when it occurs is often associated with pain, voice change and a hard lump in the throat. It is worse in male patients over 40 (or females over 50), those with neck nodes and with nodules more than 5 cm or in patients with history of radiation exposure or autoimmune thyroiditis, (which is more common in females). Children and males have a higher risk of malignancy if they present with a thyroid nodule. Multiple endocrine neoplasia may also increase the risk of malignancy.

Clinical examination involves systemic and local neck examination looking for signs of thyroid hypo- or hyper-function and para-neoplastic syndromes. The thyroid gland, cervical nodes and retro-sternum are assessed. Clinical investigation includes biochemical testing: thyroid function test, electrolytes [including: calcium, magnesium and phosphate], thyroglobulin, calcitonin; radiological investigations include ultrasound (US), magnetic resonance imaging (MRI) and computed tomography (CT) scanning; and histo-pathological diagnosis and confirmation using fine needle aspiration cytology (FNAC) [with only a 1-10% false negative rate if ultrasound guided], core biopsy and paraffin section histopathology [1].

### Disease consideration in terms of biology

Thyroid disease is unlike squamous cell carcinoma and each pathological subtype behaves in a distinct way. Many patients will coexist with their tumours and have long-term survival with minimal intervention; hence 5-year survival figures may be inappropriate. When removing thyroid cancer, complete tumour removal is the gold standard since this reduces recurrence, allows more effective radioiodine ablation and follow-up using blood test. It must always be noted that micro-metastases, however, may occur with even the smallest tumours even with complete early removal and that thyroid cancers may skip the progression from local to loco-regional to distant metastases. In effect, they are on the cusp of the Fisher spread model of cancer spread (i.e. melanoma, leukaemia and non-solid tumours) rather than the Halstead stepwise progression paradigm (i.e. squamous cell carcinoma and some breast cancers).

These micro-metastases may lie dormant and be non-iodine avid for a considerable time before becoming active. The thought process must be rationalised regarding management since a review of the subject may suggest that many patients will succumb to other insults rather than die primarily of their disease. We all eventually will have thyroid nodules, the vast majority of which will be benign and of those that are cancerous the majority will not be the ultimate cause of death. So perhaps thyroid surveillance is more of physician's agenda rather than being fully patient-, outcome- or health-economic-orientated. Larger and longer-term studies are needed to reveal or refute this suspicion.

The adage "choose well, cut well and then get them well is especially paramount for trouble free management of these cases".

### The thyroid team

Thyroid cancer is a condition now predominately treated by Otolaryngologists (ENT surgeons) who already have a thorough knowledge of vocal fold assessment and rehabilitation as well as emergency airways management both to avoid and treat common complications should they occur. They can also adequately pre-assess the voice concerns of the patients before and after surgical treatments. General surgeons also perform the operation in the UK.

The management of thyroid cancer is usually conducted by a multidisciplinary team involving ENT and general surgeons, radiation oncologists, endocrinologists, radiologists, histopathologists, cytologists and allied-healthcare professionals (i.e. speech and language therapists, dieticians, tracheostomy nurses...etc.). Patient care is usually managed with an endocrinologist so that their endocrine-related conditions are optimised prior to surgery. The patient should ideally be euthyroid for surgery since complications may arise in hypo- or hyperthyroid states. If a cancer is diagnosed, then an active involvement of a radiation oncologist (familiar with the biology of the disease and radio-iodine administration) is also required.

### The learning curve

There are few publications detailing how to deal with the difficult thyroid cancer. When compared to other cancers, it is relatively rare with several histological subtypes which run differing clinical courses and respond to different therapies.

We report on the approach to difficult thyroid in senior practice. As a consultant Otolaryngologist with an interest in thyroid surgery in South East England, this is a brief summary of the learning curve that comes from a few months of multi-disciplinary-guided practice. On MEDLINE^® ^search there was no article to date detailing how to deal with a difficult thyroid, we hope to address this need, at least partly, through our experiences and difficulties as a trainee surgeon and now as an independent consultant practitioner.

We present a breakdown of our raw results and the rules of thumb that we have gleaned from the multidisciplinary team approach, the advice of our excellent trainers and colleagues, the great and often good. When starting in this new post one of the senior consultants, kindly attended theatre to ensure the newcomer was within normal operative skill levels expected of a consultant.

This has several validatory and appraisal benefits. The experience was so mutually beneficial we often schedule operating together in our spare time or for unusual cases. We have tried our utmost to standardise our technique so that we may assess slight variances and decide what works for the individual and discard what doesn't since surgery can be a capricious mistress to even the most ardent admirer. Many things make a difficult thyroid ranging from those factors concerning the patient, his physicians and the environment.

We describe the simple hemi-thyroidectomy/lobectomy which forms the most basic operation upon which other more involved procedures are layered. We then describe the resolution strategies to several difficulties that are commonly encountered and how we as a team have negotiated them. Rhythm in surgery is important and every operation has slow and fast phases with frequent micro-pauses for observation and reflection and then action. A note of caution to the "Young Turk", although thyroid surgery may not take an excessive amount of operative time, true success is not the same as speed but outcome. A further moment or two taken to safely ensure preservation of the nerve or complete tumour resection and haemostasis is always worth it.

### Patient preparation

It is a key to operate on "euthyroid" patients who are also optimised regarding their co-morbidities, nutritional and healing status. The voice and vocal cords are checked independently and the results are noted. All patients should have preoperative imaging (US, CT and/or MRI if appropriate) and fine needle aspiration cytology; this allows operative planning including the need for neck dissection.

### Anaesthesia and special techniques

We undertake the WHO perioperative surgical check list for each patient since this gives a time for pause and reflection before a major undertaking. A good rapport must be maintained with the clinician sharing the patient's airway. We always state our intention to use nerve monitoring, any expected difficulties including superior mediastinal dissection and any expected blood loss. Our preference is to have a non-paralysed anaesthetized patient under physiologically appropriate hypotension with head raised to lower local venous pressure. During the operation we also provide continuous feedback regarding the stage of the operation and the 15 minute warning of finishing so the anaesthetist can prepare the recovery, request for the next patient and aid in final haemostasis control by raising the blood pressure to normo-tension, dropping the head of bed and performing several anaesthetic Valsava manoeuvres to raise venous pressure to help identify any potential bleeding points. If dissecting near the carotid sinus is attempted, we infiltrate local anaesthetic in the area to reduce the sudden changes in heart rate and blood pressure associated with intra-operative carotid manipulation.

### Equipment

One has to be always aware of bystander tissue effects and transmitted heating and electrical effects. In our practice, we also prefer to use magnification allowing wide field detailed recognition and examination of structures. At least 2 theatre lights are arranged to provide direct intense illumination overhead above the operative site and oblique illumination from the feet side to allow exploration of the superior poles. The scrub table is situated by the feet allowing full access to the head. Sterile light handles are used intra-operatively to make any fine adjustments. If the operative binocular ENT microscope is used, pre-adjust its eye pieces to the surgeon's inter-pupillary distance, set the appropriate lenses to allow a focal working distance of between 25-30 cm and sterile drape including lens protection. The binocular ENT microscope provides excellent illumination, "3D" depth perception and is useful when surgical loupes are not readily available.

In the main thyroid surgical area we use low current bipolar diathermy forceps, however if a neck dissection is also required we supplement this with a monopolar finger switch diathermy with a Teflon coated blade to reduce char coating (when dissecting laterally away from the recurrent laryngeal nerve). These are kept in a sterile quill readily available on the drapes. A self-adhesive scratch pad is placed in an easily accessible location in the sterile field. We also use an Endoclip^® ^applicator which becomes extremely useful when one has unfamiliar assistants with the routine use of at least two clips (patient side and one excision side). If necessary any named vessel is still preferably tied then cut, if large they are transfixed with an appropriate suture on a round bodied needle at proximal and distal resection ends (an initial safety ligature may also be used to prevent problems i.e. with the inferior end of the cut internal jugular vein collapsing into the superior mediastinum) prior to transection.

**Figure 1:**
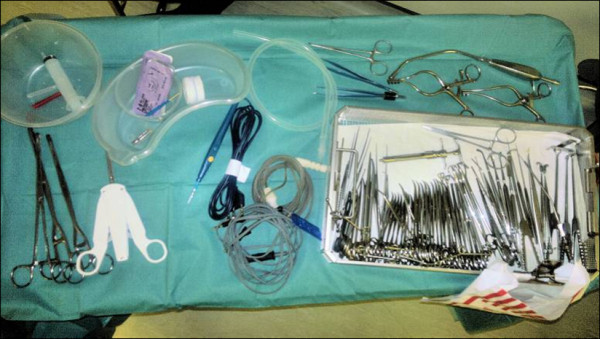
**The instrument tray**.

The appropriate vascular forceps, dissection clips and a set of extremely sharp and regular Macindoe^® ^scissors are kept on a magnetic mat on the drapes or mayo table, other sharps including needles and size 10 scalpel are only exchanged via a kidney dish placed on a flat surface (Mayo tray) with spoken acknowledgments i.e. ("scalpel to you"). Figure 1 shows the instrument tray.

### Nerve monitoring

There are many arguments for and against nerve monitoring but despite knowing the anatomy and having done this procedure many times we still use the monitor and stimulator for every case but with a healthy scepticism of the results if negative. It is not unknown for the tracheao-laryngeal electrode to become displaced and give a false negative reading, similarly the unfamiliar anaesthetist may have used a temporary neuromuscular blocking agent or the local anaesthetic used may still have an effect. We use it to confirm surgical dissection only and to acquaint the team with its benefits and pitfalls through familiarity. To not use an aid when available seems less than wise. Furthermore, we use the lowest setting once to confirm things for the briefest of time so that the team can confirm visually and audibly gross nerve continuity. Instances exist in the literature of intact stimulatable nerves that do not function on recovery. In revision cases the nerve monitor and stimulator are useful but overstimulation has its consequences.

### Operative procedure

A monitored anesthetised and appropriately consented prepared and marked patient is placed supine in extension with a sand bag under the shoulders and head controlled with a head ring. Moderately hypotensive anaesthesia without paralysis is requested. A nerve monitor and stimulator are used routinely to confirm anatomy. The skin is prepared with aqueous Betadine^® ^(unless allergic in which case chlorhexidine^® ^is used). A very dilute methylene blue infusion aids in the surgery to initially help identify the parathyroid sand then later thyroid tissue both primary and later metastatic. Perioperative intravenous steroid (8 mg dexamethasone) is used to reduce the incidence of neuropraxia. Routine antibiotics are not used presently. The table is rotated slightly to the operative side with head adjusted to place the operative field at elbow height. The patient is appropriately draped to allow an un-encumbered sterile operative field. An appropriate skin incision is marked (ideally in a skin crease) and the area infiltrated with local anaesthetic with adrenaline. After 3 minutes the incision is made and subcutaneous haemostasis is secured by bipolar diathermy, skin flaps are raised superiorly to the thyroid notch and inferiorly to the sternum mainly on the side of the operation. The platysma is incised and any superficial veins dealt with.

The anterior jugular veins if obscuring the operative field are controlled. The two Joll's retractors are placed and the operative site is fully exposed. The superficial layer of deep investing fascia is longitudinally incised and the infra-hyoid muscles meticulously delineated, often it is unnecessary to divide the sterno-thyroid muscle (which if necessary can be transected high) to allow easy access to the superior thyroid pedicle. Intelligent and sensitive retraction of a planar technique dissection field appears the key to good exposure and avoidance of complications. Vessels from the muscles to the thyroid capsule are controlled with bipolar diathermy set to below 10. Magnification is used to aid in precise and early identification of important structures. Dissection and retraction is carried out on the operative side predominately to preserve the virgin tissues of the contra-lateral side. Dissection is carried out in the line of said structures to minimise damaging them; immediate bleeding is controlled by pressure and dilute adrenaline soaked swabs before careful use of bipolar or liga-clips or fine hand ties of tissue lifted from the underling structures to minimise inadvertent damage to underlying bystander tissue. A Morris^® ^or Richardson's^® ^retractor is used to retract the medial infra-hyoid muscles laterally.

### Thyroid dissection principles

The superior pole is carefully dissected in the sterno-laryngeal or Joll's triangle (formed by the midline, infra-hyoid muscles and the superior thyroid pedicle) to identify the superior laryngeal nerve; care is taken not to damage the crico-thyroid muscle. Once identified and preserved the vessels and facial attachments of the superior pole are carefully dealt with by avoiding any mass ligation or diathermy.

Once the thyroid pole is free then an isthmusectomy is performed with a harmonic scalpel, in a superior to inferior direction based upon the anatomical attachment of the pre-tracheal fascia upon the trachea. Clips and transfixion sutures may alternatively be used to secure haemostasis.

The upper half of the thyroid is then retracted away from the thyroid bed and the inferior pole is exposed. The thyroid capsule is freed from its facial attachments high up on the substance of the thyroid to avoid any tented up nerves into the dissection field. Small vessels are evident along the capsule of the gland and appear as thin fibrous bands which can be dealt with. The recurrent laryngeal nerves are identified by pausing to observe the pulsations of the inferior thyroid artery and middle thyroid vessels. The vascular attachments are controlled. Anterior superior retraction aids identification of the nerve which is usually related to the inferior parathyroids. The inferior thyroid artery is preserved where possible and branches are divided close to the gland once the nerve is identified. This reduces devascularisation of the parathyroid glands.

The hold-up tends to be at a condensation of fascia between the anteromedial trachea and thyroid (Berry's ligament) where the nerve tents up into the operative field and may be jeopardised by inadvertent cautery or cutting. The recurrent laryngeal nerve forms one side of "Beahr's" triangle with the common carotid and inferior thyroid artery forming the other two sides.

The recurrent laryngeal nerve is reliably found by the crico-thyroid joint. Caution is also taken at the lower border of the crico-thyroid muscle near the joint to avoid the recurrent laryngeal nerve behind the superior pole. Care is taken not to macerate the muscle which may also lead to voice dysfunction or dislocate the crico-arytenoid joint.

The poor visualisation of the superior pole and superior laryngeal nerves due to the mass effect of the lesion may be alleviated by the early transection of both ipsi-lateral sterno-hyoid and thyro-hyoid muscles, however this is usually not necessary. These muscles can be cut high, since their nerve supply comes from inferiorly; their continuity is restored at the end of the operation.

**Figure 2:**
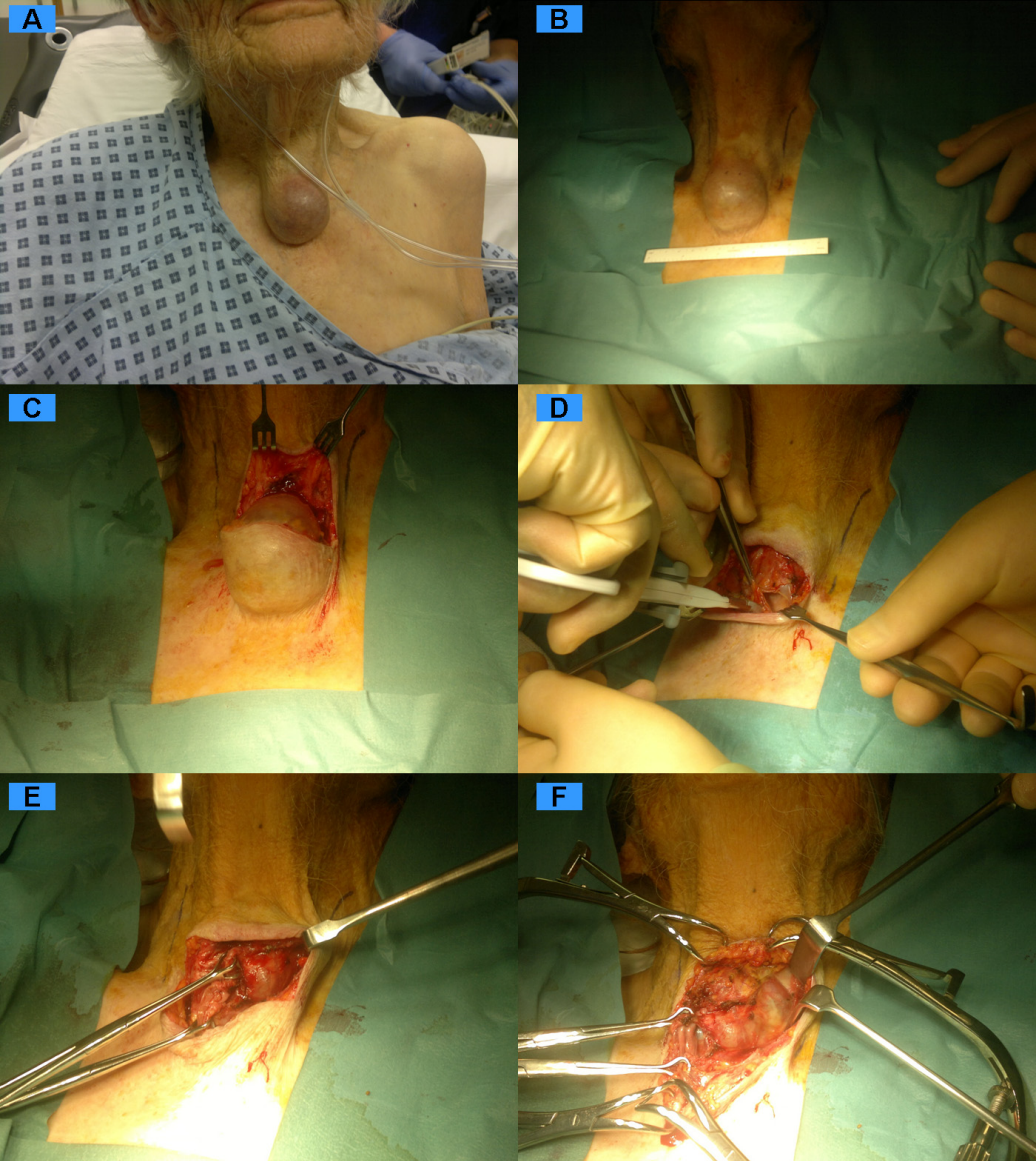
**Operative series "I" for the local anaesthetic removal of a papillary carcinoma from an unfit 84 year old lady**.

**Figure 3:**
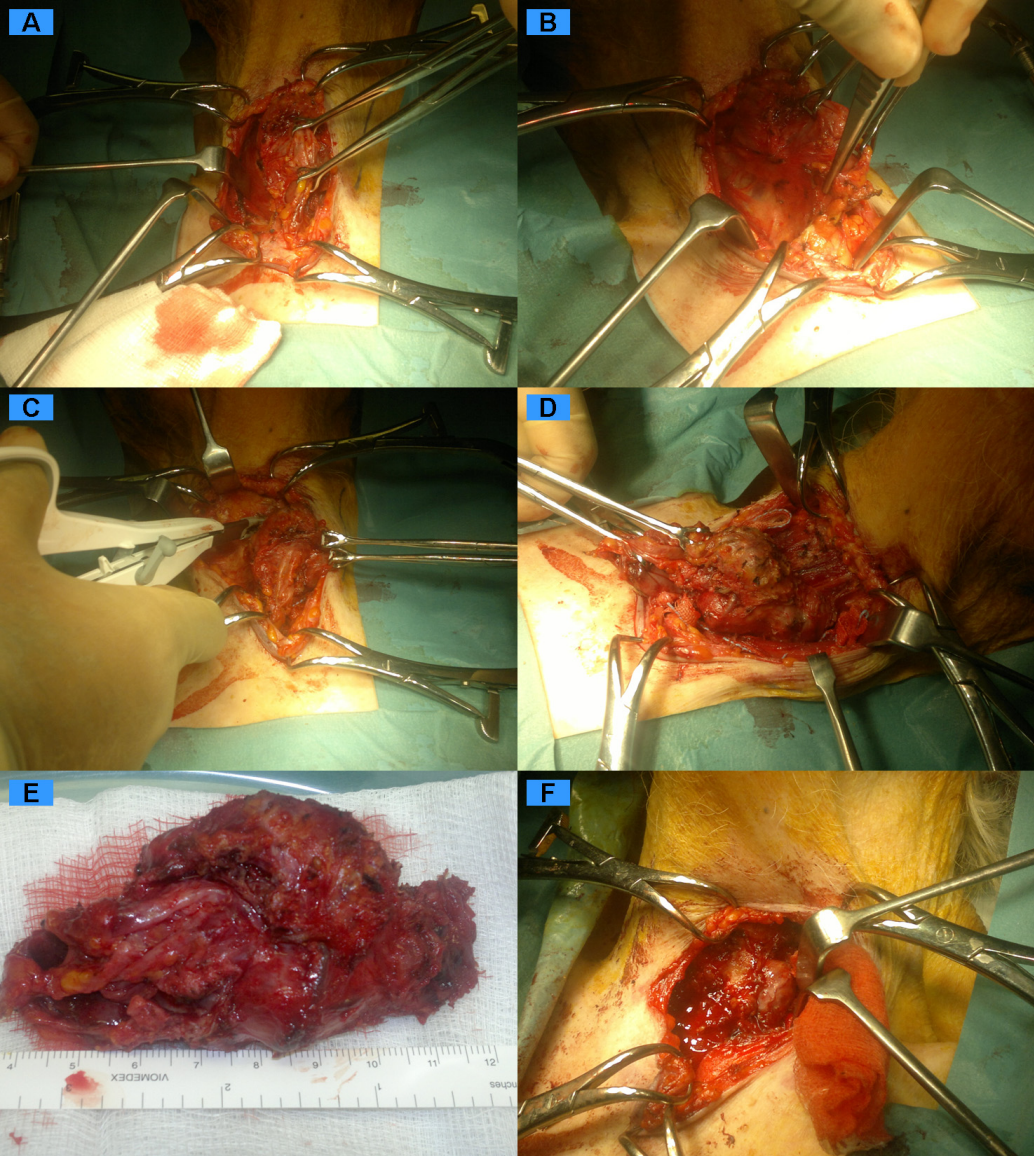
**Operative series "II" for the local anaesthetic removal of a papillary carcinoma from an unfit 84 year old lady**.

The nerve stimulator is used judiciously at a low setting to confirm anatomical structure. Only a positive stimulus correlating to anatomy is thought useful. Anatomy is the final and most dependable surgical adjunct. The identified nerves are kept within their fascial covering to minimise damage and devascularisation. Although these nerves may be robust compared to surrounding tissues in primary cases, for revision cases they must be regarded as extremely fragile and contained within variable scar tissue. Figures 2 and 3 represent operative series for the local anaesthetic removal of a papillary carcinoma from an unfit 84-year-old female.

### Difficult intra-operative situations and the common rules of thumb to overcome them

One must maintain pre-emptive and vigilant control of potential haemorrhage is logical since it doesn't require much extra skill but considerably reduces operative time and blood loss allowing a clearer operative field, reduced complications and earlier uneventful discharge. Uncontrolled bleeding from the thyroid substance is controlled by fine capsular sutures with a round bodied needle. Haemostasis is checked by head down, several anaesthetic Valsalva manoeuvres and the use of water in the wound to look for tiny bleeding sites and to help treat any theoretic cellular spillage. One must aim to leave fascial covering over the course of the nerve to avoid any direct contact with the foreign body and any attendant consequences from exposure to their breakdown products as they are metabolised. A collagen analogue dressing material (i.e. Surgicel^®^) or fascia may be placed over the course of the recurrent laryngeal nerves to protect them from the wound drain; this also aids later identification and protection of these structures if returning to theatre. However, we endeavour to minimise all foreign material within the wound.

### Surgical Drains

**Figure 4:**
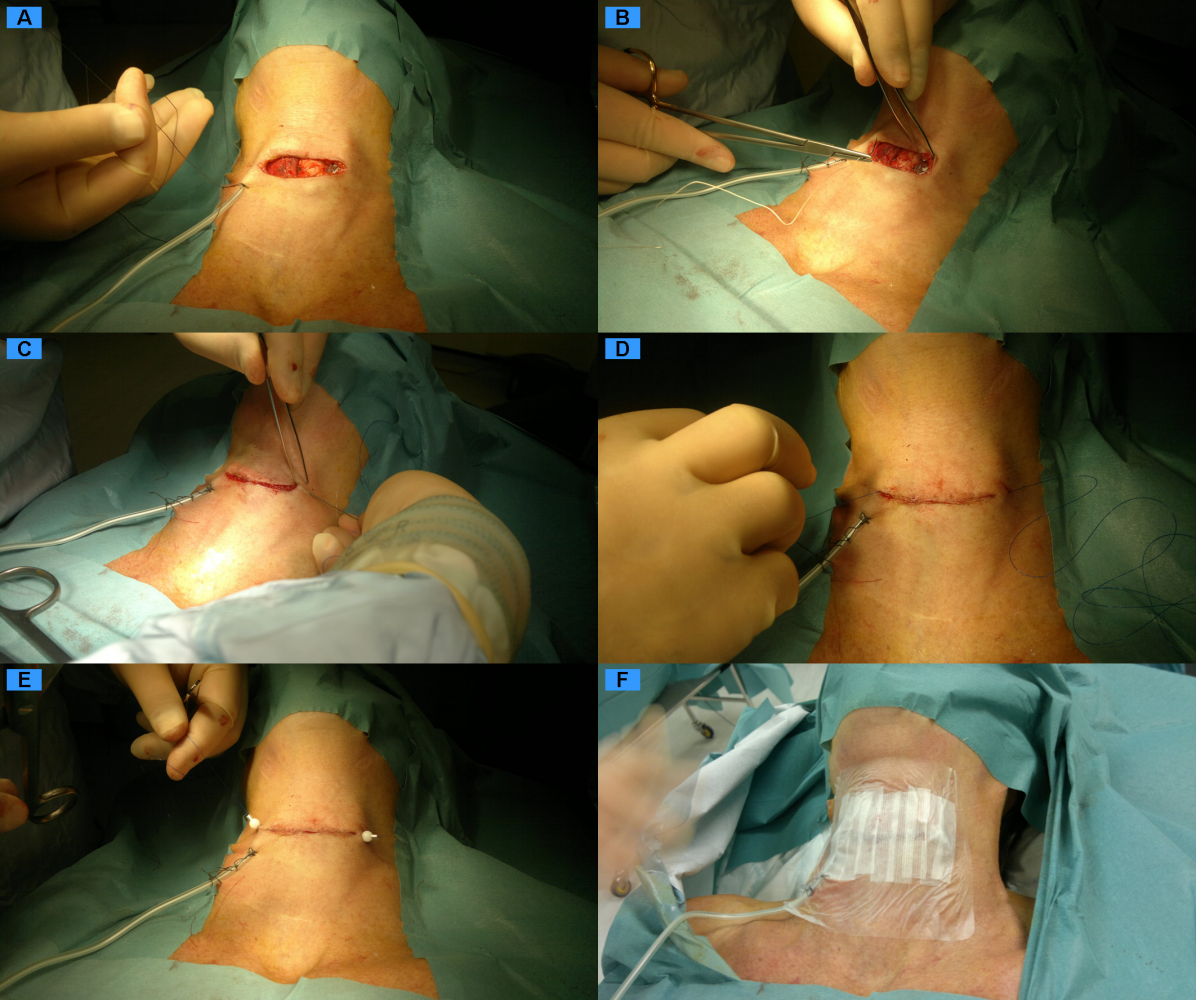
**Drain placement and skin closure**.

Suction drains are routinely used for at least 8 hours until less than 20 ml/8 hr or until drainage is minimal. Occasionally because of the limited nature of dissection, a small 10 French Haemovac^® ^or no drain may be used. Any extended procedure (i.e. with a neck dissection or in the presence of Graves, Hashimoto's or previous surgery) usually necessitates the use of a drain. Usually 16fr Redivac^® ^drains are used and are placed just lateral to the incision, they are secured with a purse string non-absorbable suture which is removed with the drain when appropriate. We try and ensure the drain path and drain holes do not lie over the bare nerves, this may necessitate some fascial approximation to bury the nerves. There are instances in the case of straightforward thyroid lobectomies where a drain need not be necessary but detailed instruction must be give for the post operative period (Figure 4).

### Closure

Before closure a warning is given to the scrub nurse who completes her instrument and swab count before handing across any suture or clipping instruments. The anaesthetist begins their preparations for recovery.

The infra-hyoid muscles if divided are re-approximated and only a few loose sutures are used to reconstitute the thyroid linear alba. The platsyma muscle is carefully re-approximated avoiding button holing of the skin. Intra-wound local acting local anaesthetic is infiltrated along the wound margins. A non-absorbable sub-cuticular suture used to close the skin without tension. Opsite^® ^spray is the used and skin tension is further reduced from the incision site by the use of vertical quarter inch steristrips^® ^across the wound. Counter pressure is occasionally placed on the neck during any stormy recovery to prevent any transmitted venous pressure rises to reduce venous haematoma rate. Otherwise the healing wound is left for observation (Figure 4).

The surgeon remains in theatre until full recovery in case of airways difficulty. An independent medical practitioner checks the vocal cord movements after the operation and makes a note. The cords are rechecked at suture removal. Any vocal fold mobility issues are discussed openly with the patient to allow early speech therapy and if necessary vocal fold rehabilitation.

### Postoperative care

The patient should be allowed to recover in an area of direct observation. Since immediate and medium term worries include airways compression by an expanding haematoma. Respiratory observation on an open ward near the nurses' station is recommended. The patient is nursed at 45 degrees and one hopes to avoid any uncontrolled forced Valsalva type manoeuvre i.e. during coughing or straining by ensuring adequate postoperative respiratory exercises, early mobilisation (VTE prophylaxis is provided for the period of immobility), if necessary open mouthed sneezing and no straining at when opening bowel.

In cases of parathyroid instrumentation or sacrifice due to tumour and in cases of prior irradiation postoperative, calcium levels are checked or supplements given in advance until the situation settles. If postoperative radioiodine ablation is contemplated, the patient is placed on short term 20 μg rT3 three times a day (with dosage adjustment as necessary). For other cases, thyroxine (T4) is started and intermittent monitoring commenced. The advice of a specialist endocrinologist is invaluable as is the postoperative multidisciplinary review of these patients.

After discharge, on return for suture removal, the steristrips^® ^are replaced for a further 3 days and the non-absorbable sutures are removed after 4-7 days. The vocal cords are independently checked and movements documented at a later clinic date.

### Special cases and caveats

One always hopes to get it right first time and experience no same 'old' mistakes just less new ones. However, mistakes or errors should be openly disclosed in a non-judgemental atmosphere so the group can learn to avoid them in the future. One's practice rapidly improves from the ignominy of admitting errors but we should always recognise and admit our losses to benefit from the reinforcement and allow everyone to get better.

The easy pole is done first since it may give you a plane for the difficult dissection which otherwise would be less apparent, similarly in total thyroidectomy the easy side is done first.

In the case of a diagnostic thyroid lobectomy but with the possibility of further contra-lateral surgery, we recommend the use of nerve monitoring, methylene blue and use a unilateral technique that doesn't jeopardise the contra-lateral dissection field to minimise subsequent bleeding and operative time and reduce complications.

For revision cases the nerve tends to be more friable and contained within solid fibrous tissue. In these cases, extremely gentle but firm pressure on instruments is required. In contrast, in virgin cases, the nerve is more robust and surrounding tissue more friable. In previous surgery, one starts inferiorly and laterally outside the previous operative field to establish anatomy and then approach the involved area.

With chances of parathyroid loss and hypocalcaemia especially in a second operation or postoperative radiotherapy (imperilling vasculature), previous operative notes and pathology must be assessed. Parathyroid tissue within the previous specimen indicates risk. Scarring or subsequent radiotherapy may have imperilled the blood supply of the remaining parathyroid tissue. Here one of the secondary aims of the operation must be to preserve viable or re-implant devitalised parathyroid tissue (locally in the sternomastoid in patients not expected to have external beam radiotherapy which may devitalise neovascularised tissue) or otherwise lead to the severe consequences of hypo-para-thyroidism.

Our practice is to try ideally to preoperatively localise residual parathyroid tissue by ultrasound and non-contrast MRI. We use methylene blue peri-operatively for all cases (in the expectation to reduce problems should we have to return to complete the thyroid). We use the different phases of parathyroid wash in/out and later take advantage of the preferential staining of thyroid tissue which reduces the differentiation from parathyroid tissue but increases that from normal bystander tissue. Extreme care is taken with the inferior thyroid artery which tends to be the main supply to the para-thyroids, hence the artery is taken as close to the gland as possible. In tumour cases the thyroid gland is not explored for the presence of parathyroid tissue. We have a unit preference of staged hemi-thyroidectomy with histo-pathological analysis. Any total thyroidectomy patient is pre-emptively commenced on short term calcium supplements after surgery to prevent postoperative hypo-calcaemia; the calcium levels are checked in a standard way and again on suture removal, this usually indicates if a further period of calcium replacement is needed; any doubt is resolved with a PTH level.

### With central neck dissection level VI and lateral neck dissections (II-V)

We use the methylene blue "wash out" phase when thyroid tissue is now stained and lymph nodes get stained aiding rapid identification of the plane and lymph node dissection reducing operative time and increasing lymph node yield. This specific usage has not been previously reported in the literature. Preoperative assessment via non-contrast MRI and US is very helpful for preoperative discussions, theatre scheduling and intra-operative localisation of the diseased lymph node levels.

Level VI neck dissection is often contemplated for completion of total thyroidectomies for malignancy and is aided by finding the recurrent laryngeal nerve in the mid to low central neck. The thymus is included in the excision specimen. Sensitivity is always given to the trachea to avoid devascularisation by surgical trauma affecting its blood supply. Any tracheomalacia may be dealt with by stenting but appears rare even in prolonged goitres.

Lateral neck dissections tend to be straight forward because of the disease biology (differing from squamous cell carcinoma) and the lack of previous chemo-radiation unless the carotid sheath is breached, in which case the internal jugular vein is commonly affected. We utilise the "wash out" phase of methylene blue intravenous administration which often stains affected lymph nodes preferentially. Perioperative ultrasound with Doppler and other cross sectional imaging may aide preoperative planning and preservation of non-involved non-obstructing anterior jugular, common facial and deep veins in one case or both internal jugular veins is lost to aid venous drainage in the head and brain to prevent rises in intracranial pressure which may be associated with its own morbidity and mortality.

In cases of loss short segment rerouting or inter-positional micro-vascular grafting may be contemplated including the use of adjunctive steroid courses and temporary airways procedures (i.e. tracheostomy) to prevent swelling and respiratory compromise. During Level IV dissections, great care is needed to avoid damaging the thoracic duct or equivalent structures because of adherent overlying nodes. If damaged we would avoid diathermy and repair with fine absorbable suture on a round bodied needle. A local muscle flap and a small piece of Surgicel^® ^may aide in the "sandwich" repair. The patient is nursed sat up at 45 degrees and straining is avoided for a few days, specialist dietetic advice is sought and any high output fistula is treated early. A discussion of the minute details of the lateral neck dissection is beyond the scope of this paper but an outline may be found in Upile et al. [2].

With massive disease, one may transect the infra-hyoid muscles since they are thinned out anyway; anatomically they are cut high to avoid cutting the supplying branches of the ansa cervicalis, since when the muscles are re-opposed they heal by fibrosis and an innervated muscle belly will still allow function.

When chasing mediastinal disease, it is essential to control the vessels in the neck; one must be aware of communicating veins between the thyroid and subclavian or brachiocephalic veins. Rigid endoscopy is a useful adjunct in these cases.

With nerve palsy of the contra-lateral side we recommend the use of nerve monitoring and commend operating with a senior colleague. We preoperatively counsel the patient and consent for potential airways intervention. We suggest the use of steroids peri-operatively to reduce the effect of any neuropraxia

### With FNA thy3 or above consider surgical risk

The interpretation of the "thy classification" can be especially confusing and multidisciplinary discussion helpful but essentially thy3 or above generally means some form of lobectomy either unilateral or bilateral. It is critical to have a specialist cytologist to help interpret results and help direct treatment based upon histo-pathological & cytological evidence. An FNA does not preclude an ultrasound-guided core biopsy which can have its own utility especially for lymphomatous processes (Table 1).

**Table 1:** thy staging and risk of cancer

thy stage	Risk of cancer
+thy3 i	20-40%
+thy3 ii rag bag term	Some risk of tumour less than i
*thy3 a	Risk 20-25% adenomatous or hyperplastic
*thy3 b	Risk 40-50%
thy4	80% risk total thyroidectomy suggested because of multifocality of disease
thy5	100% risk total thyroidectomy

*UCLH scale North Central London Thyroid Network Scale

+BAETS scale

Occasions with a nodule on the other side which is sub-T1 in size and whose risk is minimal may pose conservative options for management. One must try and avoid the temptation of total thyroidectomy.

Nodules are common and are usually benign, hence a thorough MDT discussion and frank explanation with the patient is preferred; the choices are US-guided FNA of any lesion over 0.5 cm in diameter by an experienced clinician or review of a disease process that in all probability is unlikely to reduce the long-term prognosis of the patient. The aim of good surgical decision making is whenever possible preserve natural function and form. In this case intermittent simple oral regimens of thyroxine never match the episodic and physiologically appropriate release of thyroid hormones and factors from the thyroid gland. This suggests that surgery may often be a solution to many thyroid diseases albeit a simplistic and non-physiological one.

When the disease infiltrates local structures, more radical ablative surgery may be necessary since radio-iodine may not be as effective and one must aim not to leave disease. This may necessitate tracheal shave resections, ring resection and re-anastomosis (with supra-hyoid release); sometimes total laryngectomy (especially for invasion) which may be extended, oesophageal invasion may involve partial upper oesophagectomy. Mediastinal exploration may need to be undertaken either in the form of a partial mediastinal resection to formal sternotomy to excision volume of superior mediastinal disease. One must try and preserve venous drainage on the approach since one may need to sacrifice an invaded internal jugular vein to avoid excessive postoperative oedema or raised intracranial pressure. Where this is difficult, clips may be left at operation and may help direct the oncologist in planning external beam radiotherapy.

Medullary thyroid carcinoma (MTC) is often poses several management challenges since it is a surgically treated disease which may exist within the spectrum of multiple endocrine neoplasia (MEN) and may present in the very young. During the multidisciplinary investigative phase ultrasound-guided FNA is often carried out with immuno-stains for calcitonin. Baseline TFTS, Calcitonin, PTH, calcium and vitamin D levels are taken as well as urinary 24 hour metanephrines. Genetic screening and counselling may be indicated including prophylactic surgery. Computerise tomography (CT) of neck, chest and abdomen (including liver, 3 phase contrast CT to exclude a phaechromocytoma) with further CDA testing to bench mark any systemic disease burden. The main surgery is radical especially in the young (with thymectomy) and may involve bilateral neck dissections. In an N0 neck (as shown by a high quality ultrasound by an expert radiologist) then one may perform only Level VI and VII clearances and a selective neck dissection on the same side as the lesion. A presenting nodule of 4 cm will have a 20-40% chance of ipsilateral and 20% probability of contralateral neck disease. Larger volume disease warrants bilateral selective neck dissections with level VI and VII clearance to avoid local recurrence. The 'rule of thumb' suggests that serum calcitonin greater than 15,000 is deemed incurable as is more than 3 site disease (central and two sides of the neck).

With suspicion of an anaplastic thyroid cancer, early investigation will avoid an emergency tracheostomy with a slow agonising decline. There is a tendency of an anaplastic carcinoma to grow through the stoma site; this is avoided by an ismusthectomy if feasible and if some vocal cord function and tracheal lumen is viable. The main differential is lymphoma; an ultrasound-guided core biopsy is helpful as are lesser procedures such as isthmusectomy.

The development of thyroid nodules in the young may raises the suspicion of malignancy and associated conditions (i.e. MTC), the cancer appearing more aggressive in the under 10 years of age. One must deal not only with the pathology but also the child's anxiety and parents feeling of guilt. An approach with multidisciplinary team consisting of: surgeon, paediatrician, endocrinologist, oncologist and cancer nurse specialist. Treatment decisions are made on a patient by patient basis and may range from a total thyroidectomy occasionally with radioiodine and selective neck dissections for FNA positive disease, with the follow-up being life-long.

With postoperative haematoma formation, early exploration with nerve monitoring is useful. The common areas of concern tend to be the middle thyroid veins and superior thyroid vessels but most often no focal bleeding is found but rather general ooze. A washout with warm water and gentle removal of clots with the supine patient in a head down position is helpful. Again Valsalva manoeuvres may help identify bleeding points.

## Discussion and conclusion

Good surgery involves a team effort to produce good results consistently. All members of the team are essential to quality service delivery. Communication with the team and the patient is paramount. One problem experienced by a new senior surgeon is how to consent for complications that haven't arisen but of which one wants to inform the patient. Since it may well be only a matter of chance in these cases it is often difficult to covey risk for likelihood of some complications when one hasn't experienced them yet. Estimating the risk of a rare, but plausible, serious complication when none has yet occurred is important. The non-occurrence of an adverse event in this surgical series does not mean that it cannot happen. It can, and the true rate of occurrence can be estimated from its 95% confidence interval. It is a good estimate of the worst case that is compatible with my observed data. A zero numerator does not necessarily mean zero risk. It is possible to estimate the risk of a rare plausible complication even when it has not occurred, and when only the denominator is known. This is done through the "rule of three" which states that if no major adverse events occurred in a group of n people, there can be 95% confidence that the chance of major adverse events is less than one in n/3 [3,4].

From analysis of our initial results one may conclude we are average in terms of complications from the surgical treatment of the pathology presented to us, however we will further endeavour to improve our results. This can only be done with detailed operative analysis including patient outcome measures with feedback via voice analysis pre and post operatively. We have had the benefit of a multidisciplinary preoperative opinion of our cancer cases from the North London UCLP thyroid tumour network group consisting of the surgical teams (ENT and General), endocrinologists and oncologists from at least 3 major hospitals. We have no hesitation in involving other specialists for complex cases to try and get the decisions right.

After completing our first 100 thyroid operations in senior practice we can conclude that all thyroids can be difficult thyroids be that in terms of presentation, diagnosis, patient, disease or surgical circumstances!

## Consent

Written informed consent was obtained from the patient for publication of this "how-to-do-it" review paper and accompanying images. A copy of the written consent is available for review by the Editor-in-Chief of this journal.

## Competing interests

The authors declare that they have no competing interests.

## Authors' contributions

All authors have contributed intellectually and to the writing of this manuscript. All authors read and approved the final manuscript.
